# Photochemical analysis of structural transitions in DNA liquid crystals reveals differences in spatial structure of DNA molecules organized in liquid crystalline form

**DOI:** 10.1038/s41598-018-22863-z

**Published:** 2018-03-14

**Authors:** Katarzyna Brach, Akiko Hatakeyama, Claude Nogues, Joanna Olesiak-Banska, Malcolm Buckle, Katarzyna Matczyszyn

**Affiliations:** 1 Advanced Materials Engineering and Modelling Group, Wroclaw University of Science and Technology, Wroclaw, 50370 Poland; 2 LBPA, IDA, ENS Cachan, CNRS, Université Paris-Saclay, Cachan, F-94235 France

## Abstract

The anisotropic shape of DNA molecules allows them to form lyotropic liquid crystals (LCs) at high concentrations. This liquid crystalline arrangement is also found *in vivo* (e.g., in bacteriophage capsids, bacteria or human sperm nuclei). However, the role of DNA liquid crystalline organization in living organisms still remains an open question. Here we show that *in vitro*, the DNA spatial structure is significantly changed in mesophases compared to non-organized DNA molecules. DNA LCs were prepared from pBluescript SK (pBSK) plasmid DNA and investigated by photochemical analysis of structural transitions (PhAST). We reveal significant differences in the probability of UV-induced pyrimidine dimer photoproduct formation at multiple *loci* on the DNA indicative of changes in major groove architecture.

## Introduction

It has been shown that even very short DNA duplexes, which according to the Onsager hard rod model lack the anisotropic shape required for liquid crystal ordering, are able to organize into mesophases^1^. In such mesophases, DNA chains align in parallel and because of end-to-end adhesion, form columnar aggregates. Nakata *et al*.^1^ suggested that under appropriate conditions suitable for chemical ligation of short dsDNA oligomers, the liquid crystalline phase favoured longer DNA molecule formation. Furthermore, LC DNA phases appear to be crucially involved in DNA packaging mechanisms enabling DNA to be condensed in very small volumes^2,3^. In turn, DNA packaging and its organization directly influence gene expression by modulating interactions with transcription factors^4,5^. LC arrangements may have a specific impact by placing linearly distant DNA segments in close spatial proximity. The liquid crystalline assembly of DNA chains could be a satisfactory compromise between the need for the DNA to occupy minimal space in a cell whilst simultaneously maintaining an elastic organization of the DNA^6^. However, there are no reports concerning potential gene regulatory functions of DNA liquid crystalline organization. Consequently, given the existence of DNA LC phenomena *in vivo*^3,7–12^, it is important to analyse specific models bearing a resemblance to the natural DNA concentration conditions and arrangement. Analysis of the local DNA conformation at base pair resolution is technically difficult, especially *in vivo*. Existing high-resolution methods such as X-ray crystallography and NMR cannot, to date, be applied to the study of DNA organisation in the complexity of living cells.

PhAST is a technique used for investigating protein-DNA interactions^13,14^ and is high resolution, quantitative, rapid, non-perturbing, and applicable both *in vivo* and *in vitro*^13–20^. We applied PhAST to analyse local DNA conformations in LC. Briefly, irradiation of DNA at 266 nm induces photochemical reactions, mainly leading to the formation of 5–6 cyclobutane pyrimidine (Y-Y) dimers, 6-4 photoproducts and their Dewar valence isomers and, in the presence of proteins, protein–DNA crosslinks^13^, whose yield is determined by the local conformation of DNA and its sequence. Primer extension is used to identify the position of UV induced Y-Y dimers as originally described^13^ but modified as described in^14^. Primer extension by a DNA polymerase is terminated at n-1 with respect to the first base 3′ of the Y-Y dimer on the template strand. The UV laser irradiation conditions are essentially single hit; quantification of termination, carried out by fluorescent labelling and capillary electrophoresis as described in^14^ therefore allows determination of the probability of Y-Y dimer formation at base pair resolution under a very broad range of conditions. The major product of irradiation is the formation of Y-Y dimers between adjacent pyrimidines and this clearly implies changes in the major groove. Indeed a correlation has been established between the probability of Y-Y dimer formation and the change in roll angles between adjacent bases on the same strand^14^.

The primary aim of our work was to investigate the difference between spatial DNA structure in an isotropic solution and in a liquid crystalline phase under different conditions *in vitro* (see Table 1). A characterisation of concentrated solutions of DNA and structural changes of DNA molecules in condensed phases *in vitro* is an important prerequisite for a better understanding of the complex biophysical phenomena of DNA condensation and its function *in vivo*. With PhAST technique, we were able to catalogue changes in dimer photoreactivity of LC DNA and non-organised DNA in solution under different pH and cation composition conditions. Polarized light microscopy was also used to characterize liquid crystalline phases exposed to short times of UV irradiation.

**Table 1:** List of prepared samples.

Liquid crystal		Solution	
LC1	DNA in H_2_O	S1	DNA in H_2_O
LC2	DNA in 10 mM Tris pH 7.2	S2	DNA in 10 mM Tris pH 7.2
LC3	DNA in 10 mM Tris pH 7.2, 1 mM NaCl	S3	DNA in 10 mM Tris pH 7.2, 100 mM NaCl
LC4	DNA in 10 mM Tris pH 7.2, 1 mM MgCl_2_	S4	DNA in 10 mM Tris pH 7.2, 100 mM MgCl_2_
LC5	DNA in 10 mM Tris pH 7.2, 1 mM MgCl_2_, 1 mM NaCl	S5	DNA in 10 mM Tris pH 7.2, 100 mM MgCl_2_, 100 mM NaCl
LC6	DNA in 10 mM Tris pH 7.2, 1 mM MgCl_2_, 10 mM NaCl	S6	DNA in 10 mM Tris pH 7.2, 100 mM MgCl_2_, 1 M NaCl

The indicated concentrations of Tris, MgCl_2_ and NaCl are those in solution, before the formation of liquid crystal. We considered that the increase in concentration of these elements with respect to those calculated in solution is 100 fold due to solvent evaporation.

## Results

DNA was thoroughly purified to remove potential contaminations such as RNA, proteins or organic solvents. Consequently, the effects we observed are related only to changes in DNA molecules and not influenced by interactions with other macromolecules such as proteins.

**Figure 1:**
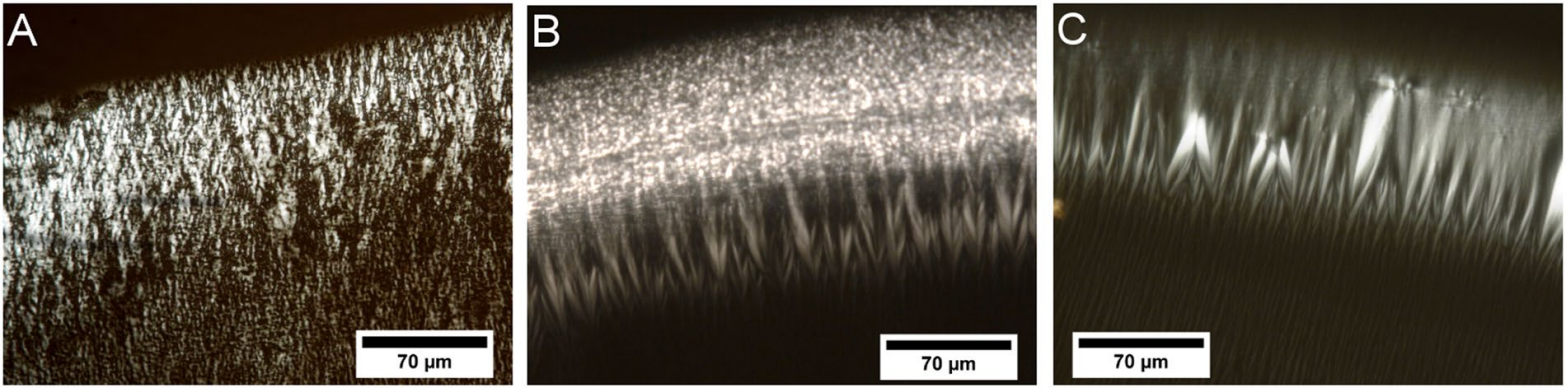
Polarized light microscopy observation of LCs. DNA LC texture (**A**) at the perimeter of a dried drop for DNA in water (LC1); (**B**) at the perimeter of a dried drop for DNA in 10 mM Tris at pH 7.2 (LC2) and (**C**) at the perimeter of a dried drop for DNA in 10 mM Tris at pH 7.2 and 1 mM MgCl_2_ (LC4).

LC ordering of supercoiled plasmid DNA has been already reported^21,22^. In our study, we used LCs obtained from linearized plasmid DNA. Initially, we prepared a series of DNA LCs placed on thin cover glasses. Liquid crystalline phase formation occurred following capillary flow in a drying droplet of DNA caused by contact line pinning. Thus, homogenously spread DNA molecules were transported from the interior to the border of the drying drop and arranged perpendicular to the contact line; this consequently led to LC forming on the edge of the droplets^23–25^. Polarized light microscopy confirmed the existence of LCs (Fig. 1). A characteristic zigzag pattern, as expected, formed at the perimeter of the dried DNA droplet (Fig. 1A). Thick and easily visible by polarized light microscopy, a liquid crystalline phase layer was present in each sample that was then used for further experiments. Additionally, microscopic examination revealed the presence of a thin DNA LC film in the interior of the dried drop. The observed texture was similar to that at the border, but was less well defined. The zigzag motif was also visible under the microscope and was arranged in small randomly distributed domains. We investigated the influence of cations (Mg^2+^ and Na^+^) and buffer (Tris pH 7.2) on LC formation. We observed that maintaining constant pH and the addition of cations significantly improved zigzag pattern formation (Fig. 1B,C). The periodicity of the zigzag pattern depends mainly on the initial volume of the droplet and the concentration of DNA and has shown to be a monotonously increasing function of the thickness of the deposit^23^. However, in our experiments, we used identical conditions (droplet volume and DNA concentration) for sample preparation. Consequently, the only differences we examine are due to pH and cations, which are known to have a significant impact on DNA conformation. Moreover, changes in ionic strength, cation composition and pH are known to modify the physicochemical properties of DNA solution (such as surface tension^26^ and contact angle) and this could directly influence the formation and periodicity of zigzag pattern. As claimed by I. Smalyukh *et al*. there are also additional factors which have to be taken into account while considering modifications of zigzag pattern periodicity (evaporation rate, viscosity, Marangoni forces, convection and capillary flow)^23^. Thus, whilst the effect of pH and cations on the periodicity of zigzag pattern formation is complex (including dynamic local changes in drying droplet, e.g. local concentration of DNA or salt), we decided to examine the effects of a subset of these factors using PhAST.

We used PhAST to analyse whether DNA LC self-assembly had a direct influence on the local DNA spatial conformation. In general, in each experiment, primer extension was carried out on DNA in solution and DNA LC under equivalent conditions and either with or without UV light irradiation. DNA conformations in LCs were compared to those in solution containing one hundred fold more salt than solutions used to prepare the LCs (Table 1) since the salt concentration was concentrated at least one hundred-fold during LC formation, due to solvent evaporation and the subsequent decrease in sample volume. Primer extension was carried out on one strand of sample DNA. We mainly focused on the region of pBSK DNA shown in Supplementary Fig. S1, although in principal the analysis could be applied indiscriminately across the whole of the DNA.

The irradiated samples yielded multiple, easily visible peaks in capillary electrophoretograms (Supplementary Figures S2A,B), while non-irradiated controls resulted only in a peak corresponding to the end of the linearized DNA fragment (Supplementary Figures S2C,D). Almost all of the termination sites in DNA solution and DNA LC were consistent with terminations preceding two or more adjacent pyrimidine residues. This result confirmed that signals obtained from irradiated DNA were related to photochemical modifications induced in the DNA by UV light.

The frequency of primer extension termination at a specific position of a sequence is related to the probability of Y-Y dimer formation at that position. The amplitude of the peak for each position is therefore directly related to the probability of Y-Y dimer formation.

PhAST should allow us to assess whether DNA LC self-assembly has had a direct influence on the local DNA groove spatial conformation because a correlation has already been observed between photoreactivity and local DNA groove architecture^14^. Thus, we compared signals from irradiated DNA in solution and irradiated DNA LC.

**Figure 2:**
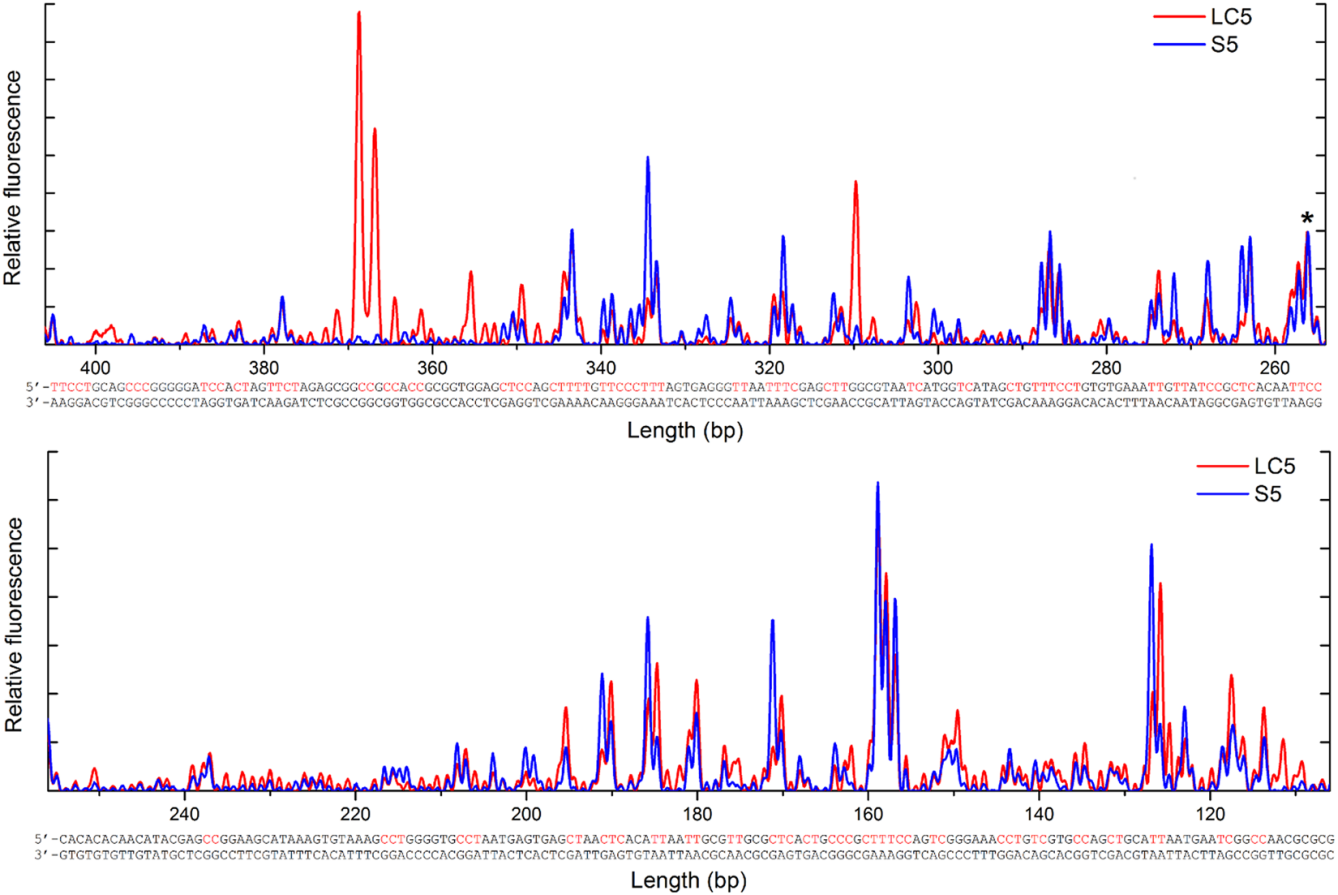
Primer extension pattern on irradiated DNA in solution S5 and liquid crystal LC5 phase after normalization, combined with the DNA sequence. Possible pyrimidine dimer formation regions are shown in red on the top strand. The same peak at the position 257 indicated with an asterisk was used for normalization in all analysed samples.

Signals obtained from irradiated DNA in LC were normalized against signals obtained from irradiated DNA in solution (example shown for comparison between LC5 and S5, Fig. 2). The inverted abscissa numbering of DNA fragments length on the graphs represents the increasing lengths of primer extension reaction products. Irradiation of DNA liquid crystals resulted in the modification of the primer extension pattern (Fig. 2, red curves). Two types of changes in signals were observed. The intensity of certain signals from irradiated DNA LC increased remarkably compared to those from irradiated DNA solution, while at the same time other signals decreased significantly. It should be noticed that there were also particular DNA regions, where peak heights were not (or only slightly) affected by DNA liquid crystalline organization. Additionally, in the case of LC samples, we observed the appearance of new peaks, not present in the case of irradiated DNA solutions, especially at positions 366 and 369, which corresponded to the formation of C-C dimers (Fig. 2). We did not notice any significant disappearance of peaks in the case of LCs.

**Figure 3:**
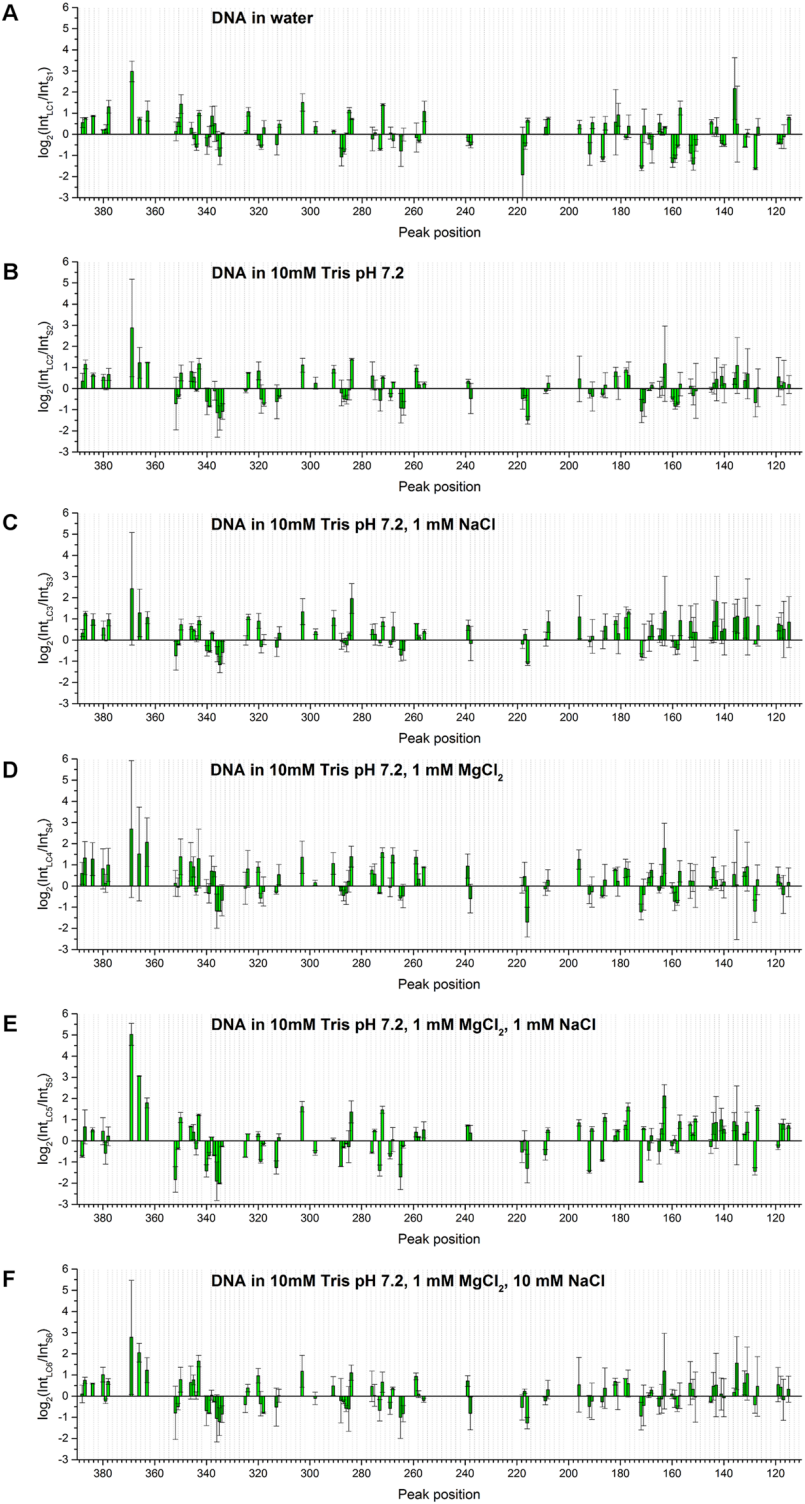
Comparison of log_2_(Int_LC_/Int_S_) obtained for (**A**) LC1 compared with S1; (**B**) LC2 compared with S2; (**C**) LC3 compared with S3; (**D**) LC4 compared with S4; (**E**) LC5 compared with S5 and (**F**) LC6 compared with S6. Average values of log_2_(Int_LC_/Int_S_) from two separate measurements are shown with standard deviation as the error. Conditions given on the graphs represent liquid crystalline samples along with the corresponding solution samples.

It is clear that liquid crystalline organization induces changes in the spatial structure of DNA molecules, leading to positive or negative changes in roll angles indicative therefore of changes in major groove architecture as suggested in^14^. As described in^14^, a more general overview of all the changes taking place on the DNA can be obtained by comparing log_2_(Int_LC_/Int_S_), where Int_LC_ is the value of a peak height observed in the case of primer extension pattern on irradiated DNA LC and Int_S_ is the same as Int_LC_, but obtained from primer extension patterns on irradiated DNA in solution. This parameter was calculated for each peak obtained with primer extension after data normalization (Fig. 3). In the case of DNA LCs, there was considerable modification of the photoreactivity compared to DNA in solution regardless of the presence of ions or buffer. In general, most of the peaks showed the same tendency with regard to the increase or decrease of specific peaks. The amplitude of peaks at 271, 284, 303, 350, 363, and 384 increased compared to DNA in solution at all conditions. While the amplitude of peaks at 158, 172, and 335 decreased significantly regardless of the ionic conditions. In the presence of buffer (Fig. 3B–F), six other peaks at 177, 182, 217, 239, 257, and 339 also showed differences in signal intensity at all salt conditions. This signal pattern reflects the LC conformation of DNA and indicates that the structural changes caused by LC formation were induced over the whole observed region leading to both increases and decreases in the probability of Y-Y dimer formation.

The highest positive and negative values of the log-ratio parameter were found between LC5 and S5; signal amplitudes are 84% higher in total than those between LC1 and S1 (Table 2), indicating the differential influence of Mg^2+^ and Na^+^ ions on DNA local structure between DNA in LC and in solution, even though there was not a linear relationship with respect to ion concentration.

**Table 2:** Comparison of total signal difference between liquid crystalline and isotropic samples.

Liquid crystal/solution	Total signal differences
LC1/S1	59.17
LC2/S2	38.98
LC3/S3	42.56
LC4/S4	49.73
LC5/S5	108.84
LC6/S6	39.71

The total signal difference was calculated as the sum of square log_2_(IntLC/IntS) values at all Y-Y positions.

The nature of interactions between mono- and divalent metal cations and double stranded DNA as well as their exact influence on DNA local structure still remains poorly described due to technical limitations. However, thanks to X-ray crystallography, NMR spectroscopy and molecular modelling we know that Na^+^ and K^+^ bind preferentially in AT-rich minor grooves, probably changing the width of the groove, whereas GC-rich major grooves can be occupied by Mg^2+^ or Ca^2+^ which may induce DNA bending or helix curvature^27–32^. Depending on the sequence of DNA used in the experiments and the experimental conditions it is also possible to show the presence of Ca^2+^ ions in minor grooves^33^ or the indirect influence of Mg^2+^ ions on minor groove width by the interaction of divalent cations with phosphate groups^34^. There are also reports suggesting a sequence-dependent differential binding of monovalent ions, where K^+^ is preferentially found in the major groove at GC steps^35,36^. As can be seen, there is no agreement in the degree to which ion binding influences DNA structure and groove structure or if it is just a result of groove structure. Our results support to some extent the theory that interactions between metal cations and DNA alter the DNA structure, although more detailed studies concerning sequence-specific ion binding and its influence on structure are needed to consolidate this observation.

**Figure 4:**
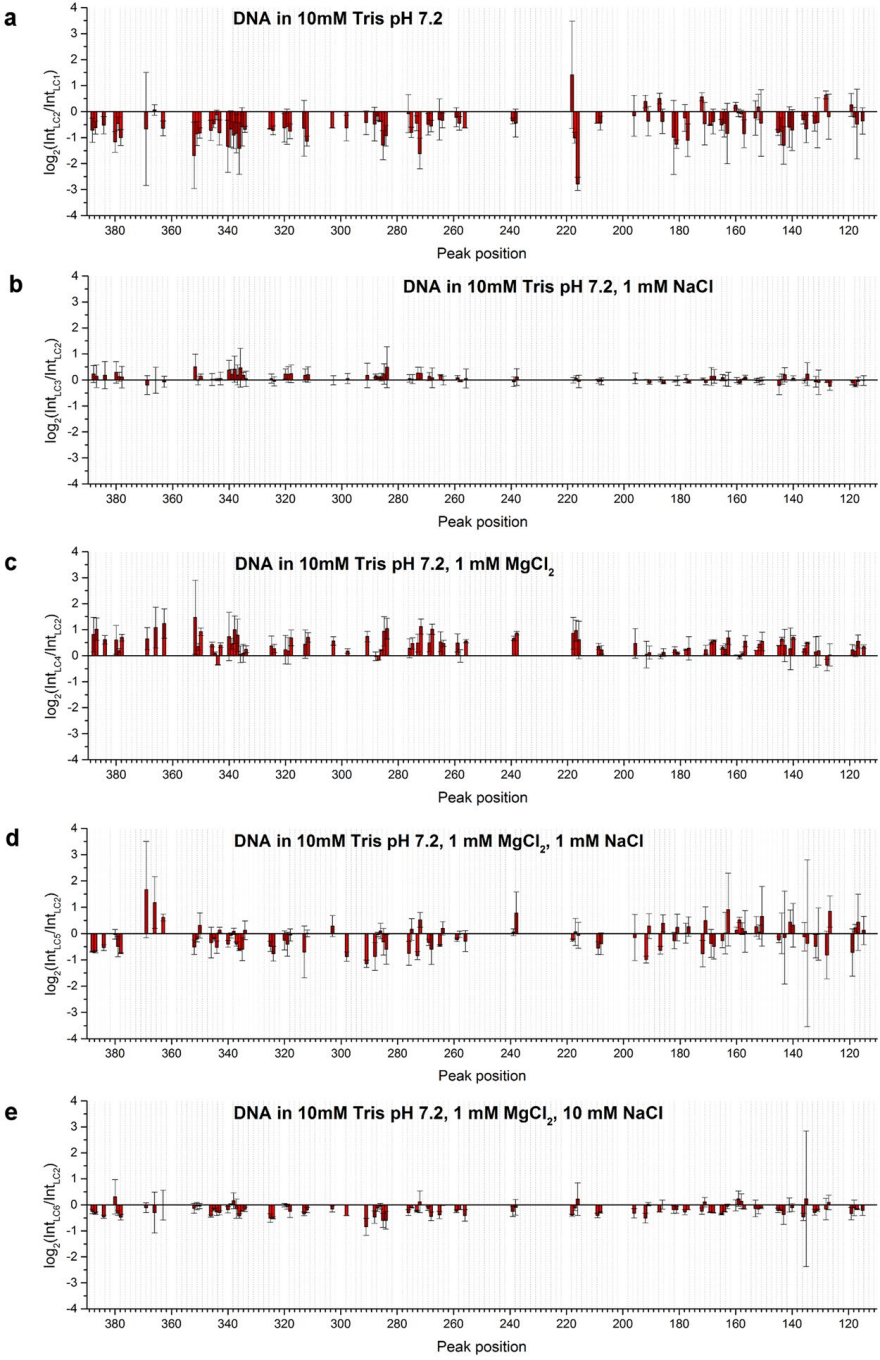
Comparison of log_2_(Int_LC_/Int_LC_) and log_2_(Int_S_/Int_S_) obtained for (**a**) LC2 compared with LC1; (**b**) LC3 compared with LC2; (**c**) LC4 compared with LC2; (**d**) LC5 compared with LC2 and (**e**) LC6 compared with LC2. Note that the scales of the Y-axis are different from those in Fig. 3.

**Figure 5:**
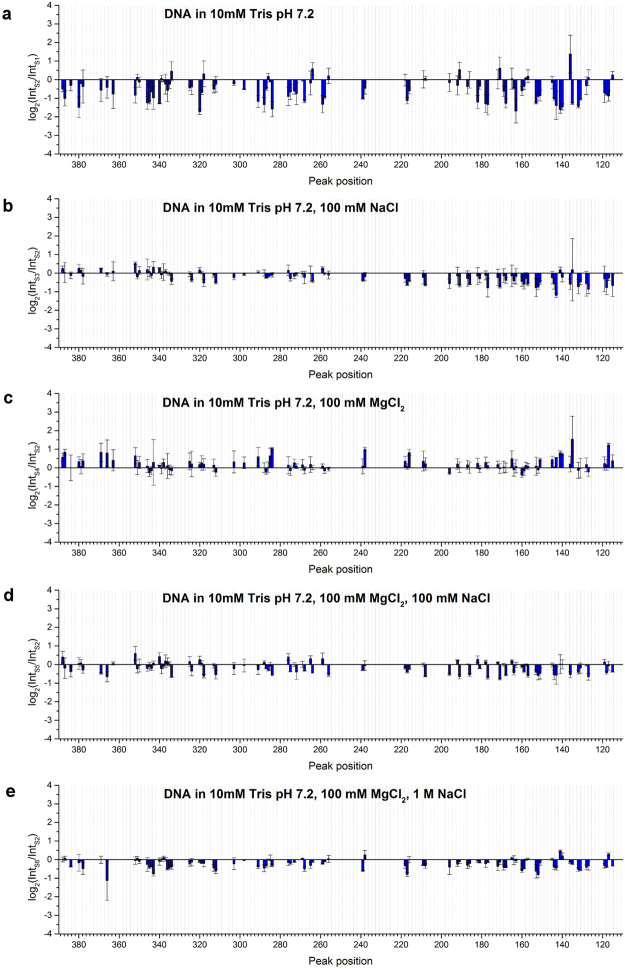
Comparison of log_2_(Int_S_/Int_S_) obtained for (**a**) S2 compared with S1; (**b**) S3 compared with S2; (**c**) S4 compared with S2; (**d**) S5 compared with S2 and (**e**) S6 compared with S2. Note that the scales of the Y-axis are different from those in Fig. 3.

To analyse the influence of ions and buffer on each state of DNA conformation, a similar analysis was carried out on DNA in LC and in solution. Results are shown in Figs 4 and 5. Signals obtained for LC and DNA in buffer, at different magnesium and/or sodium ion concentrations were compared to signals obtained in H_2_O (LC1 and S1) or in buffer (LC2 and S2), respectively.

The largest total signal difference was observed in the case of LC2 and S2 (Figs 4a and 5a), suggesting an important role of pH in the change of DNA groove architecture induced by stabilisation or destabilisation of DNA structure related to protonation of nucleic acid bases. It has been reported that pH changes dsDNA conformation and it tends to be less compact in slightly basic pH^37^. Since deionised water was slightly acidic at pH between 5.5 and 6.0, the Tris buffer (pH 7.2) is at a higher pH than water. The significant decrease we observed in the probability of Y-Y dimer formation in buffer compared to water may reflect a less compact conformation of DNA. As mentioned before, the photoreactivity of Y-Y dimer is highly sensitive to the local conformation of DNA molecules, thus any changes in DNA compaction state, as well its stability, which might be influenced by acidic pH and may lead to the structural modifications, should be also reflected in the changes of Y-Y dimer photoreactivity.

The exact nature of pH influence on DNA structure is still not well understood. It is known that protonation of bases occurs in low pH *in vitro* and that this leads to base unstacking, the protonation of GC base pairs resulting in the formation of protonated Hoogsteen base pairs and finally changes in DNA chain charge and conformational transitions of the DNA phosphate backbone^38–43^. Additionally, it was reported that mono- and divalent cations inhibit protonation of bases at low pH^40–42^. Our results showing appreciable changes in Y-Y dimer photoreactivity upon changing pH, confirm that pH significantly influences DNA structure both in solution and in LC.

The presence or absence of salts in buffer was less effective in changing the probability of Y-Y dimer formation (Figs 4b–e and 5b–e) compared to the signal differences due to the presence or absence of buffer alone. For DNA in LC, the presence of Na^+^ alone had no significant effect compared to buffer alone (Fig. 4b), suggesting that Na^+^ alone does not influence DNA conformation in LC at least as detectable by Y-Y dimer formation. On the other hand, in buffer with Mg^2+^, signals significantly increased at many locations, suggesting alterations in DNA conformation (Fig. 4c). In the presence of both Na^+^ and Mg^2+^, signals slightly decreased over the whole region observed. These results indicate that both mono- and divalent cations together affected DNA conformation in LC. In contrast, for DNA in solution, all conditions with Na^+^ showed a similar pattern of signal changes (Fig. 5b,d and e). The presence of Mg^2+^ alone increased the signal intensity at some locations (Fig. 5c). However, Mg^2+^ was less effective in changing the DNA conformation in the presence of Na^+^. Na^+^ modified the DNA conformation regardless of the presence or absence of Mg^2+^ and independent of the concentration of Na^+^ at least in the range that we tested.

In the case of DNA in LC and DNA in solution in the presence of buffer containing only magnesium ions (LC4 and S4), we observed significant increases in peak heights corresponding to an increase of roll angles. This result is strongly supported by a previous study combining an analysis of crystallographic structures and simulations showing alterations in rolls around the regions where divalent cations interact in the major groove^31^.

Values for the log-ratio parameter changed at least 26% less in total in the case of DNA in LCs compared to DNA present in isotropic solutions, indicating a lower effect of specific values of cationic ion concentration on DNA structure in the LC (Table 3). However, in the case of the comparison between LC4/LC2-S4/S2 and LC5/LC2-S5/S2 we observed at least 33% higher values of total signal difference for liquid crystalline samples. This observation reveals a subtle interplay between pH and cation concentration that may combine to induce considerable changes in DNA conformation.

**Table 3:** Comparison of total signal difference between LCs and solutions in the presence of buffer and at different ionic strengths and magnesium and sodium concentrations to signals obtained in H_2_O alone for LC1 and S1 or in buffer for LC2 and S2, respectively.

Liquid crystal/liquid crystal	Total signal differences	Solution/solution	Total signal differences
LC2/LC1	45.04	S2/S1	60.72
LC3/LC2	0.95	S3/S2	13.43
LC4/LC2	26.88	S4/S2	13.46
LC5/LC2	15.89	S5/S2	11.91
LC6/LC2	6.89	S6/S2	11.11

The total signal difference was calculated as the sum of log_2_(IntLC/IntLC) or log_2_(IntS/IntS) values at all Y-Y positions.

In conclusion, the PhAST technique can be applied to investigate the impact of liquid crystalline organization on the local conformation of DNA. The influence of Na^+^ and Mg^2+^ on DNA conformation was correlated with the type of DNA organisation.

## Discussion

The question of chromatin organization within nuclei at the chromosomal level remains open, we can therefore try to make a tenuous speculation about the relevance of our observations in this context. Reports in the literature provide some information about the cholesteric liquid crystalline type organization of dinoflagellate chromosomes^12^, stallion spermatozoa chromatin^9^ and chromosomal fibres in eutherian sperm nuclei^7^. It is reasonable to suspect that DNA in natural conditions in living cells cannot be regarded as being in a simple liquid. The proposal of Livolant *et al*.^44,45^ concerning local liquid crystalline organization of chromatin in eukaryotic nucleus being able to respond quickly to small changes in the surroundings connected with different stages of the cell cycle is extremely attractive. Thus, the modification of DNA spatial structure induced by the liquid crystalline phase could have a direct influence on gene-regulatory mechanisms. Bent DNA is known not only to facilitate transcription activation^46–48^, but is also required for protein binding to DNA^49,50^ and is involved in mismatch recognition and specificity for DNA repair^51–53^. We propose that the liquid crystalline form of DNA in living cells could serve as one of the factors controlling the accessibility of specific DNA fragments to gene-regulatory DNA-binding proteins and that liquid crystalline DNA may play a more important structural role and not simply be a result of the high local DNA concentration present in cells.

In the present study, the application of the PhAST technique that is highly sensitive to small structural changes has enabled us to show that the liquid crystalline organization of DNA has a distinct impact on the local conformation of DNA molecules. This type of DNA organization, depending on the DNA sequence and experimental conditions, leads to changes in major groove architecture. Since the regulation of gene expression is also dependent on the spatial structure of DNA chains, we suggest that, if DNA molecules are arranged locally into LCs in the living cell, the liquid crystalline organization of DNA could be a factor playing a crucial role in gene regulation. Additionally, the differential response of the photochemical reactivity of LC DNA to changes in cation concentration compared to DNA in solution suggests an intriguing role of LC organization in buffering changes in these parameters in the cell. Hence, further research with regard to the potential role of liquid crystalline organization of DNA is required to fully understand the phenomena of DNA self-arrangement and its function *in vivo*.

## Methods

### pBSK plasmid DNA isolation

Colonies of *Escherichia coli* containing pBSK plasmid DNA were streaked out onto an antibiotic-selective Luria Bertani (LB) agar plate. A starter culture of 10 mL LB medium containing ampicillin (100 μg/mL) was inoculated with a single colony picked from the plate and grown at 37 °C for 8 h with vigorous shaking (approx. 300 rpm). 5 mL of the starter culture was added into 2.5 L of LB medium and incubated at 37 °C overnight with continuous shaking. The bacterial cells were harvested by centrifugation at 6000 g for 15 min at 4 °C. The cell pellet was re-suspended in 125 mL of an ice-cold resuspension buffer (50 mM Tris-Cl, pH 8.0; 10 mM EDTA; 100 μg/mL RNase A), and then 125 mL of a lysis buffer (200 mM NaOH, 1% SDS w/v) was added and incubated at room temperature for 5 min in order to lyse the bacterial cells. After the lysate became viscous, 125 mL of a chilled neutralization buffer (3.0 M potassium acetate, pH 5.5) was added to precipitate genomic DNA. The solution was quickly mixed, left on ice for 30 min and then centrifuged at 17000 g for 30 min at 4 °C. The supernatant layer was removed and centrifuged again as described above. Further isolation of pBSK plasmid DNA was carried out using QIAGEN Plasmid Giga Kit according to the manufacturer’s handbook. The isolated plasmid DNA was dissolved in 4.5 mL of TE buffer (1 mM EDTA pH 8.0; 10 mM Tris, pH 7.4) and 450 μL of FastDigest® buffer and 10 μL of FastDigest® EcoRI were added to the DNA solution. The isolation process yielded 12.88 mg of pBSK plasmid DNA.

### Phenol/chloroform purification and ethanol precipitation

A UV-Vis spectroscopic analysis of aqueous pBSK solution was done to determine the DNA purity and concentration. The isolation process resulted in high DNA contamination. A routine protocol for purifying and precipitating DNA was used^54^. The phenol/chloroform extraction was repeated twice. The DNA was then precipitated with absolute ethanol and rinsed twice with 70% ethanol. The purification and precipitation step produced 9.81 mg of plasmid DNA. The wavelength of maximum absorption (λ_max_) for purified DNA was at 260 nm. The ratio of the absorbance at 260 and 280 nm (A_260/280_) was 1.93.

### LC preparation and microscopy observation

10 μL of each DNA aqueous solution (C_DNA_ = 0.5 mg/mL, LC1–DNA dissolved in water, LC2–DNA dissolved in 10 mM Tris pH 7.2, LC3–DNA dissolved in 10 mM Tris pH 7.2 and 1 mM NaCl, LC4–DNA dissolved in 10 mM Tris pH 7.2 and 1 mM MgCl_2_, LC5–DNA dissolved in 10 mM Tris pH 7.2, 1 mM MgCl_2_ and 1 mM NaCl, LC6–DNA dissolved in 10 mM Tris pH 7.2, 1 mM MgCl_2_ and 10 mM NaCl) was deposited on a thin, clean cover glass and left to air-dry. Characteristic liquid crystalline zigzag patterns formed on the edge of the drying droplet^23–25^. DNA LCs were observed using an Olympus 60BX polarized light microscope.

### PhAST

20 μL of each DNA aqueous solution (C_DNA_ = 50 ng/μL, S1–DNA dissolved in water, S2–DNA dissolved in 10 mM Tris pH 7.2, S3–DNA dissolved in 10 mM Tris pH 7.2 and 100 mM NaCl, S4–DNA dissolved in 10 mM Tris pH 7.2 and 100 mM MgCl_2_, S5–DNA dissolved in 10 mM Tris pH 7.2, 100 mM MgCl_2_ and 100 mM NaCl, S6–DNA dissolved in 10 mM Tris pH 7.2, 100 mM MgCl_2_ and 1 M NaCl) was put in a protein low binding 0.5 mL Eppendorf tube and exposed to around 30 mJ single pulse of 266 nm UV laser beam (Quanta-Ray INDI-40-10 10 pulsed Nd:YAG laser with a repetition rate of 10 Hz). In order to get optimal signals, DNA LCs were irradiated with a single or multiple UV laser pulses (equivalent to an energy of around 30 mJ/pulse), and were then collected from the glass surface by dissolving LCs in 10 μL of distilled water. According to the literature^13^, increasing the number of laser pulses does not cause a noticeable change in the number of UV-induced DNA lesions. In this study we used a DNA reverse primer fluorescently labelled with 6-FAM at the 5′ end R1090 (5′-CCGCCTTTGAGTGAGCTGATA-3′). The irradiated and non-irradiated samples were subjected to primer extension using Taq polymerase (0.5 U), dNTPs (0.2 mM), Taq polymerase buffer (1×), each primer (0.02 mM), respectively, and distilled water. The final reaction volume was 20 μL. The primer extension cycle consisted of denaturation at 95 °C for 6 min, primer annealing at 55 °C for 1 min and extension at 72 °C for 9 min. DNA was precipitated then with absolute ethanol and washed with 70% ethanol. DNA pellets were re-suspended in a 10 μL solution containing 9.75 μL highly deionized formamide and 0.25 μL GeneScan™ 600 LIZ® dye Size Standard. Following sample denaturation at 95 °C for 5 min, primer extension products were analysed by automated capillary electrophoresis using Applied Biosystems 3500 Genetic Analyser. Electrophoretograms and fragment analysis were performed with the GeneMapper® software v4.1 as described in^14^.

## Electronic supplementary material

Supplementary information
