# Supplementary material for: Photochemical analysis of structural transitions in DNA liquid crystals reveals differences in spatial structure of DNA molecules organized in liquid crystalline form
Source: Sci Rep. 2018 Mar 14;8:4528. doi: 10.1038/s41598-018-22863-z (PMC5852169; doi:10.1038/s41598-018-22863-z)
Supplement: Supplementary file 1 — Supplementary information [file 41598_2018_22863_MOESM1_ESM.pdf]

SUPPLEMENTARY INFORMATION

Photochemical analysis of structural transitions in DNA liquid crystals reveals differences in spatial structure of DNA molecules organized in liquid crystalline form

Katarzyna Brach, Akiko Hatakeyama, Claude Nogues, Joanna Olesiak-Banska, Malcolm Buckle*, Katarzyna Matczyszyn*

```
         10        20        30        40        50        60        70        80        90       100
      *    *    *    *    *    *    *    *    *    *    *    *    *    *    *    *    *    *    *    *
3'-GGCGGAAACTCACTCGACTATGGCGAGCGGCGTCGGCTTGCTGGCTCGCGTCGCTCAGTCACTCGCTCCTTCGCCTTCTCGCGGGTTATGCGTTTGGCGG

         110       120       130       140       150       160       170       180       190       200
      *    *    *    *    *    *    *    *    *    *    *    *    *    *    *    *    *    *    *    *
   AGAGGGGCGCGCAACCGGCTAAGTAATTACGTCGACCGTGCTGTCCAAAGGGCTGACCTTTCGCCCGTCACTCGCGTTGCGTTAATTACACTCAATCGAG

         210       220       230       240       250       260       270       280       290       300
      *    *    *    *    *    *    *    *    *    *    *    *    *    *    *    *    *    *    *    *
   TGAGTAATCCGTGGGGTCCGAAATGTGAAATACGAAGGCCGAGCATACAACACACCTTAACACTCGCCTATTGTTAAAGTGTGTCCTTTGTCGATACTGG

         310       320       330       340       350       360       370       380       390       400
      *    *    *    *    *    *    *    *    *    *    *    *    *    *    *    *    *    *    *    *
   TACTAATGCGGTTCGAGCTTTAATTGGGAGTGATTTCCCTTGTTTTCGACCTCGAGGTGGCGCCACCGCCGGCGAGATCTTGATCACCTAGGGGGCCCGA-5'
```

Figure S1. Part of pBSK sequence analyzed during our experiments. The complementary sequence for R1090 reverse primer is in blue. Y-Y steps in the region analyzed (bases between 110-400) are shown in red.

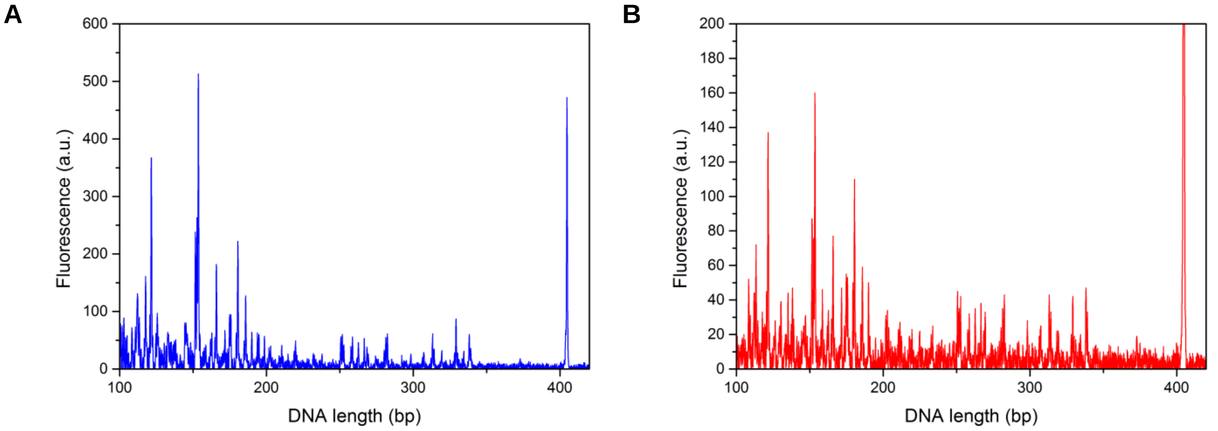

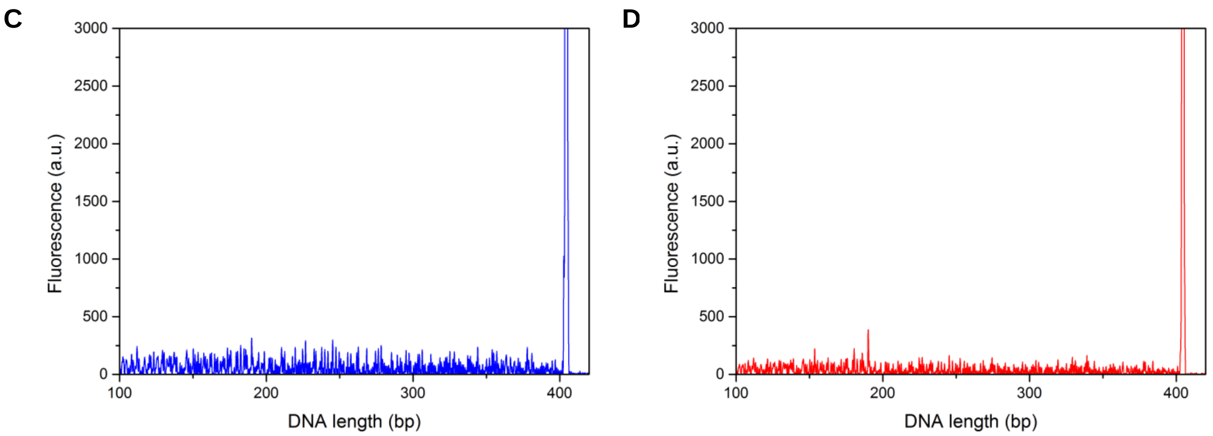

Figure S2. Differences between capillary electrophoretograms obtained after primer extension with R1090 primer for (A) irradiated DNA solution S1; (B) irradiated DNA LC1; (C) non-irradiated DNA solution S1 and (D) non-irradiated DNA LC1.
